# The prevalence of depressive symptoms in Chinese longevous persons and its correlation with vitamin D status

**DOI:** 10.1186/s12877-018-0886-0

**Published:** 2018-08-29

**Authors:** Yao Yao, Shihui Fu, Hao Zhang, Nan Li, Qiao Zhu, Fu Zhang, Fuxin Luan, Yali Zhao, Yao He

**Affiliations:** 1 Institute of Geriatrics, Chinese PLA General Hospital, Fuxing Road 28, Beijing, 100853 People’s Republic of China; 2 Beijing Key Laboratory of Aging and Geriatrics, Chinese PLA General Hospital, Beijing, China; 3 National Clinical Research Center for Geriatrics Diseases, Chinese PLA General Hospital, Beijing, China; 4 Department of Geriatric Cardiology, Chinese PLA General Hospital, Beijing, China; 5 Department of Cardiology, Hainan Branch of Chinese PLA General Hospital, Sanya, China; 6 Department of health policy and management, Texas A&M University, College Station, USA; 7 Department of Geriatric Endocrinology, Chinese PLA General Hospital, Beijing, China; 8 Central Laboratory, Hainan Branch of Chinese PLA General Hospital, Jianglin Road 9, Sanya, 572000 People’s Republic of China

**Keywords:** Vitamin D, Depression, Longevous persons, China

## Abstract

**Background:**

Hypovitaminosis D and depressive syndromes are common conditions in old adults. However, little is known about the relationship between vitamin D and depression in exceptional aged people. The objective of this study is to evaluate the relationship between vitamin D levels and depressive symptoms in Chinese longevous persons.

**Methods:**

We used a dataset from a cross-sectional survey of a sample of Chinese longevous people with self-reported age 100 or older, including 175 men and 765 women, was conducted from June 2014 to December 2016 in Hainan Province, China. Data on demographics, lifestyle characteristics and health conditions were collected using a structured questionnaire. Anthropometrics and blood samples were obtained following the standard procedure. Depressive symptoms of the participants were assessed using a shortened version of the Geriatric Depression Scale (GDS-15). Serum vitamin D levels were measured using an automated radioimmunoassay.

**Results:**

The prevalence of longevous persons with depressive symptoms among the sample was 32.2% (95% confidence interval: 29.7–34.7%). Serum vitamin D levels were lower in participants with depressive symptoms than in those without (20.8 ± 8.7 vs. 23.7 ± 9.7, ng/mL). Vitamin D deficiency was an independent risk factor for depression after controlling for the potential covariates (Odds ratio = 1.47, 95% Confidence interval = 1.08–2.00; *p* = 0.014). A negative relationship between serum vitamin D levels and depressive symptoms was also detected, and the relationship remained significant after adjusting for a wide range of other covariates. The multivariate adjusted odds ratio of depressive symptoms for the lowest versus highest quartiles of vitamin D levels was 1.73 (95% confidence interval: 1.10–2.72), and the adjusted odds ratio with a 5 ng/mL decrement of serum 25OHD levels was 1.10 (95% confidence interval: 1.01–1.19).

**Conclusions:**

This study showed an inverse association between vitamin D levels and depressive symptoms among Chinese longevous persons. Depressive symptoms should be screened in longevous persons who had vitamin D deficiency. Further studies on vitamin D supplement and prevention along with treatment of depression are needed among very old population.

## Background

Depression is a common condition in elder population, and depressive symptoms are more prevalent in oldest-old adults [1–3]. Older adults with depressive symptoms face with numerous adverse health outcomes including functional decline, cognitive impairment, disability, decreased quality of life, and increased mortality rate from co-occurring medical conditions and suicide [4, 5].

Depression is not an essential part of aging itself [6], but oldest-old populations are more vulnerable to depressive symptoms than younger old-ones [1–3]. This vulnerability could be partly explained by age-related structural and biological changes [7]. Higher prevalence of depressive symptoms may also due to the increased health-related risk factors in later life (i.e., physical disability, cognitive impairment, social isolation, institutionalization, and bereavement) [8]. However, the risk factors of depression may vary with age [9]. There is a need, therefore, to develop assessment strategies that enhance our capacity to identify very old adults who at higher risk for depression. Vitamin D provides such an opportunity.

Vitamin D plays a critical role in physical and mental health [10]. The 25-dihydroxyvitamin D regulates more than 200 genes in the human body and is responsible for musculoskeletal and neural health [11]. Inadequate vitamin D levels in later life could lead to a higher risk of falls, fractures, cardiovascular diseases, diabetes and other comorbid conditions, all of which could cause mental disorders [12, 13]. In addition, vitamin D has been reported to play a neuroprotective role through several physiological mechanisms such as calcium homeostasis, neurogenesis, antioxidant defense, immunomodulation, and amyloid beta clearance [14–16].

A few studies that analyzed the association between vitamin D status and depressive symptoms have yielded equivocal results [17–21]. Vidgren et al. reported that a lower vitamin D levels is associated with a higher prevalence of depression in elderly general population aged 53 years and older [22]. Jovanova et al. found that vitamin D levels are cross-sectionally but not prospectively associated with depression in late-life population aged 55 and older [23]. Zhao et al. claimed that no significant association between serum concentrations of 25(OH)D and the presence of moderate-to-severe depression, major depression or minor depression was found among US adults [18]. Besides, data on vitamin D and depressive symptoms in elderly Asian population are scarce, particular for Chinese longevous persons. Therefore, the aim of this study was to address the dearth of evidence on vitamin D levels and depressive symptoms in Chinese longevous persons. It is hypothesized that, in accordance with the evidence of vitamin D’s neuroprotective role, longevous persons with lower concentrations of serum 25(OH)D would have greater odds of depressive symptoms.

## Methods

### Participants and setting

The sample of this study was obtained from the China Hainan Centenarian Cohort Study (CHCCS), which was conducted in Hainan, China from June 2014 to December 2016. Details of this study including sampling strategy and interview procedures have been described elsewhere [24, 25]. In brief, a total of 1002 longevous persons with self-reported age 100 or older (180 men and 822 women) were interviewed and blood samples were collected in this study. Sixty-two participants were excluded from the data analyses because of missing information on depressive symptoms and relevant variables. The final analyses included 940 longevous persons, of which 175 were men and 765 were women. Surveys on longevous persons need rigorous age validation, and the accuracy of the age reporting is essential [26]. The longevous persons in CHCCS were identified according to the household register provided by the civil affairs bureau of Hainan in 2014, and the age verification process was conducted before the participants were included in the study.

Written informed consent was obtained from all men and women who participated in the CHCCS study. The Ethics Committee of the Hainan branch of the Chinese People’s Liberation Army General Hospital (Sanya, Hainan) approved the study protocol (No. 301hn11201601).

### Measurements

#### Depressive symptoms

Depressive symptoms were measured using the shortened version of the Geriatric Depression Scale (GDS-15), which is a self-administered screening tool developed to detect depression in older adults [27]. The responses to the 15 dichotomous items of this scale were scored and higher scores indicate more depressive symptoms (possible range 0–15; observed range of 0–15). As previously described, a score of more than 6 points was considered to have a depressive symptom [28].

#### Vitamin D levels

Serum circulating 25-hydroxyvitamin D, often abbreviated as 25(OH)D, is commonly used as a measurement of vitamin D status [29]. Blood samples were obtained from each participant by an experienced nurse and transported in cold storage to the Clinical Laboratory and were assayed within 4 h. Serum 25(OH)D concentrations were measured by automated radioimmunoassay analyzers (DiaSorin, Stillwater, MN, USA) using a standard procedure. The inter-assay and intra-assay coefficients of variation for 25(OH)D in the present study were 8.3% and 6.7%, respectively. Vitamin D deficiency was defined as 25(OH)D < 20 ng/mL or 50 nmol/L according to the Endocrine Society Clinical Practice Guidelines [30].

#### Covariates

Home interviews were conducted to collect demographic data (age, sex, ethnicity, education, living conditions, and social interactions), lifestyle characteristics (smoking, alcohol use, tea drinking, milk drinking, fish intake, and fruit consumption), activities of daily livings (ADLs), outdoor activities (daily outdoor walking or gardening for more than 1 h), and common conditions (visual and auditory impairments) of the participants. Ethnicities were dichotomized into Han versus non-Han. Given that the majority of longevous persons received no education, participants were dichotomized into illiterate versus primary school or above. Participants were asked whether they were living with families or not for living conditions. Social interactions included relatives and friends contacts, which were defined as meet and communicate with at least one relative or friend once a month. ADLs of the participants were scored according to Barthel Index (BI), and a score of less than 90 was regarded as ADL impairments [31]. Habits of smoking, alcohol drinking, and tea drinking were dichotomized into current versus former or never. Frequent milk, fish, and fruit intakes referred to consumption at least once per week.

Anthropometrics including heights and weights were measured by well-trained nurses following the standard procedures. Body mass index (BMI) was calculated as the weight in kilograms divided by square height in meters. Systolic and diastolic blood pressures (SBP and DBP) were measured two times consecutively, with at least 1-min interval between measurements, and the reported blood pressures were the average of the two measurements. Samples of venous blood were obtained from the centenarians in a seated position for at least 5-min rest and transported in cold storage (4 °C) to Central Laboratory within 4 h. Serum concentrations of triglyceride (TG), total cholesterol (TC), fasting blood glucose (FBG), albumin (ALB), hemoglobin (Hb) and creatinine were measured using enzymatic assays (Roche Products Ltd., Basel, Switzerland) on a fully automatic biochemical autoanalyzer (COBAS c702; Roche Products Ltd). Estimated glomerular filtration rate (eGFR) was calculated using a modified version of Modification of Diet in Renal Disease (MDRD) equation based on the data from Chinese patients as follows: 175 × serum creatinine (mg/dL)^− 1.234^ × age (year)^− 0.179^ × 0.79 (if female) [32]. All assays were performed by qualified technicians without knowledge of clinical data.

### Statistical analyses

Continuous variables were described as the mean and standard deviation for variables with normal distribution and the median and interquartile range for variables with skewed distribution. Categorical variables were described as the percentage. Continuous variables were compared using Student’s *t* test (normal distribution) and Mann–Whitney *U* test (skewed distribution). Multivariate logistic regression analyses were performed to determine the independent correlates of depression symptoms. Odds ratios (OR) and 95% confidence intervals (CI) of depressive symptoms were further evaluated according to quartile of serum 25(OH)D concentrations and one 5-ng/mL decrement of serum 25(OH)D concentrations using logistic regression analyses. Adjustments were made for age, sex, ethnicity, education, living conditions, social interactions, lifestyles, ADL impairments, outdoor activities, BMI, SBP, DBP, TG, TC, FBG, ALB, Hb, estimated GFR, visual impairments, and auditory impairments. A test for linear trend across serum 25(OH)D concentrations was performed by assigning median values of serum 25(OH)D levels for each quartile. Statistical significance was accepted at the two-sided 0.05 level, and CI was computed at the 95% level. Statistical analyses were performed with Statistic Package for Social Science (SPSS) version 19.0.

## Results

A total of 940 longevous individuals were included in this study with self-reported age ranging between 100 and 115. As Table 1 shows, the majority participants were female (81.4%), Han ethnic (87.9%), illiterate (91.0%), and living with families (85.7%). Of the participants, nearly 44% of them met and interacted with relatives at least once a month, and one third of them socialized with friends. Only 3% of the participants were current smokers, and about 11% and 12% of them were current alcohol drinkers and tea drinkers, respectively. The proportions of frequent milk drinking, frequent fish intake, and frequent fruit consumptions were 26.3%, 63.5%, and 58.9%, respectively. More than half of the participants had ADL impairments and 60% of the participants had at least one hour outdoor activities a day. Twenty-eight percent of individuals had a visual impairment, and 31% had an auditory impairment.

**Table 1:** Characteristics of the participants according to depressive symptoms

Characteristics	Total Sample, *n* = 940	Depression, *n* = 303	No Depression, *n* = 637	*P*
Serum vitamin D, ng/mL, mean ± SD	22.7 ± 9.5	20.8 ± 8.7	23.7 ± 9.7	< 0.001
Vitamin D deficiency, %	39.8%	47.9%	35.9%	< 0.001
Age, mean ± SD	102.5 ± 2.7	102.5 ± 2.8	102.5 ± 2.7	0.802
Females, %	81.4%	89.8%	77.4%	< 0.001
Han ethnic, %	87.9%	87.5%	88.1%	0.432
Illiterate, %	91.0%	95.7%	88.7%	< 0.001
Living with families, %	85.7%	84.8%	86.2%	0.618
Relatives contacts, %	43.7%	39.9%	51.8%	0.001
Friends contacts, %	33.7%	32.0%	37.3%	0.121
BMI, kg/m^2^, mean ± SD	18.3 ± 3.2	17.8 ± 3.3	18.5 ± 3.1	0.004
SBP, mmHg, mean ± SD	152.1 ± 24.7	152.9 ± 25.5	151.7 ± 24.4	0.504
DBP, mmHg, mean ± SD	75.5 ± 13.2	75.2 ± 13.7	75.7 ± 12.9	0.538
TG, mmol/L	1.17 ± 0.67	1.18 ± 0.83	1.16 ± 0.58	0.615
TC, mmol/L, mean ± SD	4.66 ± 1.04	4.58 ± 1.05	4.70 ± 1.04	0.105
FBG, nmol/L, mean ± SD	4.87 ± 1.88	5.00 ± 1.77	4.81 ± 1.92	0.131
ALB, g/L, mean ± SD	38.4 ± 4.1	37.8 ± 4.2	38.7 ± 4.0	0.002
Hb, g/L, mean ± SD	113.2 ± 16.8	110.5 ± 17.3	114.4 ± 16.3	0.001
Estimated GFR, ml/min per 1.73m^2^, mean ± SD	75.8 ± 28.5	77.1 ± 29.2	75.2 ± 28.2	0.358
Current smoker, %	3.5%	3.3%	3.6%	0.488
Current alcohol drinker, %	10.6%	8.6%	11.6%	0.096
Current tea drinker, %	12.1%	11.2%	12.6%	0.318
Frequent milk drinking, %	26.3%	29.0%	25.0%	0.205
Frequent fish intake, %	63.5%	62.4%	64.1%	0.664
Frequent fruit intake, %	58.9%	61.7%	57.6%	0.256
ADL impairments, %	56.4%	72.3%	48.8%	< 0.001
Outdoor activities > 1 h/d, %	59.6%	52.5%	63.0%	0.001
Visual impairments, %	28.0%	28.4%	27.8%	0.454
Auditory impairments, %	31.2%	30.4%	31.6%	0.386

Abbreviation: *SD* standard deviation, *BMI* body mass index, *SBP* systolic blood pressure, *DBP* diastolic blood pressure, *TG* triglyceride, *TC* total cholesterol, *FBG* fasting blood glucose, *ALB* albumin, *Hb* hemoglobin, *GFR* estimated glomerular filtration rate, *ADL* activity of daily living

**Fig. 1:**
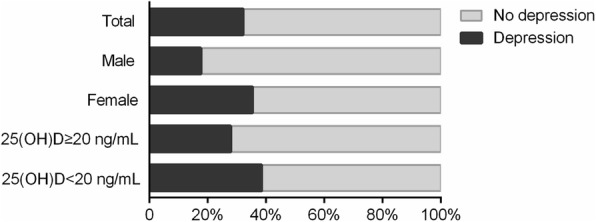
Percentages of participants with depressive symptoms by gender and vitamin D status. Differences in proportions were tested using Chi-square test. Overall prevalence of depressive symptoms was 32.2% (95% confidence interval, 29.1–35.1%). Females had higher prevalence of depressive symptoms than males (35.6% vs. 17.7%, *P* <  0.001). Participants with vitamin D deficiency had higher prevalence of depressive symptoms than those without (38.8% vs. 27.9%, *P* <  0.001)

The mean ± SD of GDS-15 scores was 5.3 ± 3.2, and the overall prevalence rate of the depressive symptoms among the longevous persons was 32.2% (95% CI, 29.7–34.7%). The mean ± SD of serum 25(OH)D levels was 22.7 ± 9.5 ng/mL, and 39.8% of the participants were vitamin D deficient (< 20 ng/mL). Females (35.6% vs. 17.7%), and those with vitamin D deficiency (38.8% vs. 27.9%) were more likely to have depressive symptoms than males and those without vitamin D deficiency (*p* <  0.001 for all, Fig. 1).

Table 1 compares the characteristics of participants with and without depression. In addition to sex, participants who had a depression were more likely to be illiterate and were less likely to have relatives contacts (*P*s <  0.001). No statistically significant differences were found in lifestyle variables. The prevalence of ADL impairments was higher in participants who had a depression than those who did not (*P* <  0.001). Participants without depression were more likely to participate in regular outdoor activities (*P* = 0.001). Serum concentrations of 25(OH)D, albumin, hemoglobin and BMI were significantly lower in participants with depression than those without (*P*s <  0.01).

Table 2 shows the results from the multivariate logistic regression analyses. Participants who were deficient in vitamin D, female, illiterate, not living with families, had no relative contacts, or had ADL impairments tended to have higher odds of depression (*P*s <  0.05). The adjusted OR between vitamin D deficiency and depressive symptoms after adjusting for other covariates was 1.47 (1.08–2.00; *P* = 0.014).

**Table 2:** Significant associates for depressive symptoms by multivariate logistic regression

Characteristics	β	OR	95% CI	*P*
Vitamin D deficiency	0.386	1.47	1.08–2.00	0.014
Age	0.020	1.02	0.97–1.08	0.470
Females	0.600	1.82	1.10–3.03	0.021
Han ethnic	−0.156	0.86	0.53–1.39	0.529
Illiterate	0.927	2.53	1.26–5.05	0.009
Living with families	−0.541	0.58	0.38–0.90	0.015
Relatives contacts	−0.501	0.61	0.45–0.82	0.001
Friends contacts	−0.189	0.83	0.60–1.15	0.255
BMI	0.028	1.03	0.98–1.08	0.251
SBP	−0.004	1.00	0.99–1.00	0.266
DBP	0.003	1.00	0.99–1.02	0.652
TG	−0.163	0.85	0.67–1.07	0.165
TC	0.123	1.13	0.96–1.33	0.146
FBG	−0.040	0.96	0.89–1.04	0.320
ALB	0.006	1.01	0.96–1.05	0.785
Hb	0.009	1.01	1.00–1.02	0.100
eGFR	−0.001	1.00	0.99–1.00	0.708
Current smoker	−0.468	0.63	0.26–1.52	0.300
Current alcohol drinker	0.239	1.27	0.72–2.23	0.407
Current tea drinker	−0.127	0.88	0.54–1.44	0.615
Frequent milk drinking	−0.124	0.88	0.62–1.25	0.487
Frequent fish intake	0.002	1.00	0.73–1.38	0.991
Frequent fruit intake	0.049	0.95	0.69–1.32	0.767
ADL impairments	0.944	2.57	1.81–3.65	< 0.001
Outdoor activities > 1 h/d	−0.011	0.99	0.71–1.37	0.948
Visual impairments	0.170	1.19	0.81–1.73	0.376
Auditory impairments	0.032	1.03	0.72–1.49	0.864

Abbreviation: *β* logistic regression coefficient, *OR* odds ratio, *95% CI* 95% confidence interval, *BMI* body mass index, *SBP* systolic blood pressure, *DBP* diastolic blood pressure, *TG* triglyceride, *TC* total cholesterol, *FBG* fasting blood glucose, *ALB* albumin, *Hb* hemoglobin, *eGFR* estimated glomerular filtration rate, *ADL* activity of daily living

To further investigate the correlation between vitamin D levels and depressive symptoms in longevous persons, the participants were categorized into four groups according to the quartiles of serum 25(OH)D concentrations. As Table 3 shows, a negative correlation between serum 25(OH)D and depressive symptoms was observed. After adjusting for confounding variables, this negative relationship remained statistically significant. The multivariable adjusted odds ratio of depressive symptoms for the lowest versus highest quartiles of vitamin D levels was 1.73 (95% CI, 1.10–2.72). When comparing the 2nd and 3rd quartiles with the highest quartile, the odds ratio of depressive symptoms were 1.61 (1.04–2.50) and 1.09 (0.69–1.71), respectively (*P* for trend = 0.003). In addition, the multivariable adjusted OR between depressive symptoms and one 5-ng/mL decrement of serum 25OHD level was 1.10 (1.01–1.19, *P* <  0.001).

**Table 3:** Odds ratios of depressive symptoms according to the quartile of serum vitamin D levels

Variable	Quartile 1, *n* = 235	Quartile 2, *n* = 231	Quartile 3, *n* = 236	Quartile 4, *n* = 238	*P* for Linear Trend	5-ng/mL Decrement of Plasma Vitamin D
Serum vitamin D, ng/mL, mean (range)	12.0 (3.9–16.3)	19.2 (16.4–21.5)	24.4 (21.6–28.0)	35.1 (28.1–65.1)		
Depression, % (n)	41.3% (97)	38.5% (89)	26.7% (63)	22.7% (54)		
Unadjusted OR (95% CI)	2.48 (1.67–3.70) ^***^	2.02 (1.35–3.03) ^**^	1.24 (0.82–1.89)	1.00	< 0.001	1.20 (1.10–1.29) ^***^
Adjusted OR^a^ (95% CI)	1.73 (1.10–2.72) ^*^	1.61 (1.04–2.50) ^*^	1.09 (0.69–1.71)	1.00	0.003	1.10 (1.01–1.19) ^*^

Abbreviation: SD, standard deviation; OR, odds ratio; 95% CI, 95% confidence interval

^a^Adjusted for age, sex, ethnicity, education, living conditions, social interactions, BMI, SBP, DBP, TG, TC, FBG, ALB, Hb, eGFR, lifestyle variables, ADL impairments, outdoor activities, visual impairments, and auditory impairments

*P* < 0.05^*^, 0.01^**^, 0.001^***^

## Discussion

In the current study, we investigated the association between vitamin D status and depressive symptoms in a population-based sample of Chinese longevous persons in Hainan Province. Low vitamin D levels were associated with a decreased prevalence of depressive symptoms, and this association remained unchanged after adjusting for demographic and other potential confounders. Based on this Chinese study with relatively large sample size, we added to the evidence of the association between vitamin D status and depression among long-lived persons.

Both vitamin D deficiency and depressive symptoms are common conditions in elder population [33]. Previous studies have provided evidence that vitamin D had neuroprotective roles and was beneficial to reduce the risks of mental disorders, such as depressive symptoms [12, 17, 19]. In this study, lower vitamin D levels were found to be associated with greater adjusted odds (odds ratio = 1.75) of depressive symptoms. This result is in accordance with previous studies. Chan revealed that serum 25(OH)D was inversely associated with depression in older men in Hong Kong, China [34]. Jaaskelainen reported that higher serum 25(OH)D concentrations were related to a reduced risk of depression in a representative sample of Finnish population aged from 30 to 70 [17]. Milaneschi has reported that older adults who had lower 25(OH)D levels had significantly higher risk of developing depressive mood over the 6 years follow-up (hazard ratio: 2.0) [33].

The mechanism through which vitamin D plays a beneficial role in mental health has not been clearly understood. The bioactive form of vitamin D, calcitriol, regulates calcium concentrations of neurons by intra- and extracellular atmosphere, consequently reducing toxicity caused by excessive calcium [15, 35]. Calcitriol also up-regulates glutathione metabolism in neurons, thus promoting antioxidants activities that protect them from the processes of oxidative degeneration [36]. In addition, vitamin D promotes the expression of nerve growth factor and stimulates neurogenesis [35]. Other studies have revealed that Vitamin D had a role in amyloid beta clearance [15]. Moreover, it has been reported that vitamin D regulates gene expression of tyrosine hydroxylase, an essential enzyme involved in the synthesis of norepinephrine and dopamine [37]. Norepinephrine and dopamine are two neurotransmitters involved in mood regulation and depressive symptoms.

Vitamin D deficiency is highly prevalent in very old population worldwide [4]. Numerous cost-effective intervention strategies including vitamin D supplement, fortified food intake, sunlight exposure have been identified as efficient ways in correcting vitamin D deficiency [30, 38]. Prevention of vitamin D deficiency may become a future strategy to prevent the development of depressive symptoms [39]. In addition, normalization of vitamin D levels may become a part of depression treatment in the very old [33]. These hypotheses should be further tested in appropriately designed, randomized, controlled trials.

While highlighting the strengths of the present study, there are some limitations should be acknowledged. First, this study was cross-sectional designed, and thus causality could not be inferred. A longitudinal study on vitamin D and depressive symptoms would further illuminate the clinical significance and predictive validity of depression in the Chinese oldest-old. Second, the depressive symptoms were evaluated by the GDS-15, and the diagnoses of depression were not confirmed by clinical psychiatrists. However, the GDS-15 is a commonly used scale to measure late life depression, and it has been verified as a stable assessment of depressive symptoms in old persons [40]. Third, age exaggeration may exist among this population [26, 41]. As the current household registration system did not exist in China a hundred years ago, the age of the longevous persons was mainly self-reported and could not be well-validated because of lack of solid evidence. Further studies were clearly warranted to address the issues on age validation among this exceptional people in Hainan Province, China.

## Conclusions

In summary, this study found an inverse association between vitamin D levels and depressive symptoms in a population-based sample of Chinese longevous individuals in Hainan Province, China. The findings indicate that very old individuals with low vitamin D levels could be an important target for detection of depressive symptoms. Whether interventions of vitamin D deficiency are ideal strategies or not in preventing the development of depression or treatment of the depressive symptoms in the very old population should be validated in appropriately designed, randomized, and controlled trials.

## Abbreviations

25(OH)D: 25-hydroxyvitamin D
95% CI: 95% confidence intervals
ADLs: activities of daily livings
ALB: Albumin
BMI: Body mass index
CHCCS: China Hainan Centenarian Cohort Study
DBP: Diastolic blood pressure
eGFR: Estimated glomerular filtration rate
FBG: Fasting blood glucose
GDS-15: Geriatric Depression Scale
Hb: Hemoglobin
MDRD: Modification of Diet in Renal Disease
OR: Odds ratios
SBP: Systolic blood pressure
TC: Total cholesterol
TG: Triglyceride

## References

[1] Scheetz LT, Martin P, Poon LW (2012). Do centenarians have higher levels of depression? Findings from the Georgia centenarian study. J Am Geriatr Soc.

[2] Luppa M, Sikorski C, Luck T, Ehreke L, Konnopka A, Wiese B, Weyerer S, Konig HH, Riedel-Heller SG (2012). Age- and gender-specific prevalence of depression in latest-life--systematic review and meta-analysis. J Affect Disord.

[3] Jeon HS, Dunkle RE (2009). Stress and depression among the oldest-old: a longitudinal analysis. Research on aging.

[4] Fiske A, Wetherell JL, Gatz M (2009). Depression in older adults. Annu Rev Clin Psychol.

[5] Cullum S, Metcalfe C, Todd C, Brayne C (2008). Does depression predict adverse outcomes for older medical inpatients? A prospective cohort study of individuals screened for a trial. Age & Ageing.

[6] van't Veer-Tazelaar PJ, van Marwijk HW, Jansen AP, Rijmen F, Kostense PJ, van Oppen P, van Hout HP, Stalman WA, Beekman AT (2008). Depression in old age (75+), the PIKO study. J Affect Disord.

[7] Blazer DG (2000). Psychiatry and the oldest old. Am J Psychiatry.

[8] Stek ML, Gussekloo J, Beekman AT, van Tilburg W, Westendorp RG (2004). Prevalence, correlates and recognition of depression in the oldest old: the Leiden 85-plus study. J Affect Disord.

[9] Margrett J, Martin P, Woodard JL, Miller LS, MacDonald M, Baenziger J, Siegler IC, Davey A, Poon L, Georgia Centenarian S (2010). Depression among centenarians and the oldest old: contributions of cognition and personality. Gerontology.

[10] Holick MF, Chen TC (2008). Vitamin D deficiency: a worldwide problem with health consequences. Am J Clin Nutr.

[11] Holick MF (2007). Vitamin D deficiency. N Engl J Med.

[12] Chei CL, Raman P, Yin ZX, Shi XM, Zeng Y, Matchar DB (2014). Vitamin D levels and cognition in elderly adults in China. J Am Geriatr Soc.

[13] Chu F, Ohinmaa A, Klarenbach S, Wong ZW, Veugelers P. Serum 25-Hydroxyvitamin D concentrations and indicators of mental health: an analysis of the Canadian health measures survey. Nutrients. 2017;9(10) 10.3390/nu9101116 PMC5691732 29027946

[14] Dickens AP, Lang IA, Langa KM, Kos K, Llewellyn DJ (2011). Vitamin D, cognitive dysfunction and dementia in older adults. CNS drugs.

[15] Kalueff AV, Eremin KO, Tuohimaa P (2004). Mechanisms of neuroprotective action of vitamin D(3). Biochemistry Biokhimiia.

[16] Kesby JP, Eyles DW, Burne TH, McGrath JJ (2011). The effects of vitamin D on brain development and adult brain function. Mol Cell Endocrinol.

[17] Jaaskelainen T, Knekt P, Suvisaari J, Mannisto S, Partonen T, Saaksjarvi K, Kaartinen NE, Kanerva N, Lindfors O (2015). Higher serum 25-hydroxyvitamin D concentrations are related to a reduced risk of depression. Br J Nutr.

[18] Zhao G, Ford ES, Li C, Balluz LS (2010). No associations between serum concentrations of 25-hydroxyvitamin D and parathyroid hormone and depression among US adults. Br J Nutr.

[19] Ju SY, Lee YJ, Jeong SN (2013). Serum 25-hydroxyvitamin D levels and the risk of depression: a systematic review and meta-analysis. J Nutr Health Aging.

[20] Husemoen LL, Ebstrup JF, Mortensen EL, Schwarz P, Skaaby T, Thuesen BH, Jorgensen T, Linneberg A (2016). Serum 25-hydroxyvitamin D and self-reported mental health status in adult Danes. Eur J Clin Nutr.

[21] de Koning EJ, van Schoor NM, Penninx BW, Elders PJ, Heijboer AC, Smit JH, Bet PM, van Tulder MW, den Heijer M, van Marwijk HW (2015). Vitamin D supplementation to prevent depression and poor physical function in older adults: study protocol of the D-Vitaal study, a randomized placebo-controlled clinical trial. BMC Geriatr.

[22] Vidgren M, Virtanen JK, Tolmunen T, Nurmi T, Tuomainen TP, Voutilainen S, Ruusunen A (2018). Serum concentrations of 25-Hydroxyvitamin D and depression in a general middle-aged to elderly population in Finland. J Nutr Health Aging.

[23] Jovanova O, Aarts N, Noordam R, Zillikens MC, Hofman A, Tiemeier H (2017). Vitamin D serum levels are cross-sectionally but not prospectively associated with late-life depression. Acta Psychiatr Scand.

[24] He Y, Luan FX, Yao Y, Yang SS, Xie HG, Li J, Liu M, Wang JH, Wu L, Zhu Q (2017). China Hainan centenarian cohort study: study design and preliminary results. Chinese Journal of Epidemiology.

[25] He Y, Zhao Y, Yao Y, Yang S, Li J, Liu M, Chen X, Wang J, Zhu Q, Li X, et al. Cohort profile: the China Hainan centenarian cohort study (CHCCS). Int J Epidemiol. 2018;47(3):694-5. 10.1093/ije/dyy017 29506028

[26] Yi Z, DLP J, Vlosky DA, Gu D. Healthy longevity in China: Springer; 2009.

[27] Almeida OP, Almeida SA (1999). Short versions of the geriatric depression scale: a study of their validity for the diagnosis of a major depressive episode according to ICD-10 and DSM-IV. International journal of geriatric psychiatry.

[28] Tang WK, Chan SS, Chiu HF, Wong KS, Kwok TC, Mok V, Ungvari GS (2004). Can the geriatric depression scale detect poststroke depression in Chinese elderly?. J Affect Disord.

[29] Zerwekh JE (2008). Blood biomarkers of vitamin D status. Am J Clin Nutr.

[30] Holick MF, Binkley NC, Bischoff-Ferrari HA, Gordon CM, Hanley DA, Heaney RP, Murad MH, Weaver CM, Endocrine S (2011). Evaluation, treatment, and prevention of vitamin D deficiency: an Endocrine Society clinical practice guideline. J Clin Endocrinol Metab.

[31] Vermeulen J, Neyens JC, Rossum EV, Spreeuwenberg MD, Witte LPD (2011). Predicting ADL disability in community-dwelling elderly people using physical frailty indicators: a systematic review. BMC Geriatr.

[32] Ma YC, Zuo L, Chen JH, Luo Q, Yu XQ, Li Y, Xu JS, Huang SM, Wang LN, Huang W (2006). Modified glomerular filtration rate estimating equation for Chinese patients with chronic kidney disease. Journal of the American Society of Nephrology : JASN.

[33] Milaneschi Y, Shardell M, Corsi AM, Vazzana R, Bandinelli S, Guralnik JM, Ferrucci L (2010). Serum 25-hydroxyvitamin D and depressive symptoms in older women and men. J Clin Endocrinol Metab.

[34] Chan R, Chan D, Woo J, Ohlsson C, Mellstrom D, Kwok T, Leung P (2011). Association between serum 25-hydroxyvitamin D and psychological health in older Chinese men in a cohort study. J Affect Disord.

[35] Garcion E, Wion-Barbot N, Montero-Menei CN, Berger F, Wion D (2002). New clues about vitamin D functions in the nervous system. Trends Endocrinol Metab.

[36] Shinpo K, Kikuchi S, Sasaki H, Moriwaka F, Tashiro K (2000). Effect of 1,25-dihydroxyvitamin D(3) on cultured mesencephalic dopaminergic neurons to the combined toxicity caused by L-buthionine sulfoximine and 1-methyl-4-phenylpyridine. J Neurosci Res.

[37] Newmark HL, Newmark J, Vitamin D (2007). Parkinson's disease--a hypothesis. Movement disorders : official journal of the Movement Disorder Society.

[38] Johnson MA, Kimlin MG (2006). Vitamin D, aging, and the 2005 dietary guidelines for Americans. Nutr Rev.

[39] Young SN. Has the time come for clinical trials on the antidepressant effect of vitamin D? J Psychiatry Neurol Sci. 2009;34(1):3. PMC2612077 19125208

[40] Gana K, Bailly N, Broc G, Cazauvieilh C, Boudouda NE (2017). The geriatric depression scale: does it measure depressive mood, depressive affect, or both?. International journal of geriatric psychiatry.

[41] Gu D, Huang R, Andreev K, Dupre ME, Zhang Y, Liu H. Assessments of mortality at oldest-old ages by province in China's 2000 and 2010 censuses. Int J Popul Stud. 2016;2(2):1–25.

